# Formaldehyde exposure and leukemia risk: a comprehensive review and network-based toxicogenomic approach

**DOI:** 10.1186/s41021-021-00183-5

**Published:** 2021-04-12

**Authors:** Doo Seok Kang, Hyun Soo Kim, Jong-Hyeon Jung, Cheol Min Lee, Yeon-Soon Ahn, Young Rok Seo

**Affiliations:** 1 Department of Life Science, Institute of Environmental Medicine for Green Chemistry, Dongguk University Biomedi Campus, 32 Dongguk-ro, Ilsandong-gu, Goyang-si, Gyeonggi-do 10326 Republic of Korea; 2 Faculty of Health Science, Daegu Haany University, Gyeongsan, Gyeongbuk 38610 Republic of Korea; 3 Department of Chemical and Biological Engineering, College of Natural Science and Engineering, Seokyeong University, Seoul, 02173 Republic of Korea; 4 Department of Preventive Medicine and Institute of Occupational and Environmental Medicine, Wonju College of Medicine, Yonsei University, Wonju, Gangwon 26426 Republic of Korea

**Keywords:** Formaldehyde, Leukemia, Toxicogenomics, Biological network analysis, Carcinogenicity

## Abstract

Formaldehyde is a widely used but highly reactive and toxic chemical. The International Agency for Research on Cancer classifies formaldehyde as a Group 1 carcinogen, based on nasopharyngeal cancer and leukemia studies. However, the correlation between formaldehyde exposure and leukemia incidence is a controversial issue. To understand the association between formaldehyde exposure and leukemia, we explored biological networks based on formaldehyde-related genes retrieved from public and commercial databases. Through the literature-based network approach, we summarized qualitative associations between formaldehyde exposure and leukemia. Our results indicate that oxidative stress-mediated genetic changes induced by formaldehyde could disturb the hematopoietic system, possibly leading to leukemia. Furthermore, we suggested major genes that are thought to be affected by formaldehyde exposure and associated with leukemia development. Our suggestions can be used to complement experimental data for understanding and identifying the leukemogenic mechanism of formaldehyde.

**Supplementary Information:**

The online version contains supplementary material available at 10.1186/s41021-021-00183-5.

## Background

Formaldehyde is a colorless, pungent-smelling, and highly reactive chemical with toxic properties. As the simplest aldehyde form (H-CHO), formaldehyde is synthesized by the catalytic oxidation of methanol. It is also easily dissolved in water; a 37% formaldehyde solution is used as a preservative, pesticide, and disinfectant. Formaldehyde is manufactured commercially and used extensively in many products, such as resins, plastics, textiles, wood products, adhesives, medicines, and cosmetics [1]. The predominant route of formaldehyde exposure is inhalation occurring during environmental and occupational exposure [2]. Environmental exposure to formaldehyde occurs more frequently indoors than outdoors due to the widespread use of products containing formaldehyde [3]. The World Health Organization recommends an indoor limit of formaldehyde of 0.1 mg/m^3^ (0.08 ppm) [4]. Occupational exposure to formaldehyde is variable and occurs in numerous industries, including manufacturing [5]. Small amounts of formaldehyde are naturally generated in living organisms through normal metabolic processes, such as DNA/RNA/histone demethylation and oxidative deamination [6]. The concentration of endogenous formaldehyde in the blood of humans is approximately 2–3 mg/L (0.1 mM) [7]. Therefore, many people are constantly exposed to formaldehyde, in large or small quantities, in their daily lives because of its ubiquitous nature.

Exogenous formaldehyde exposure is commonly associated with eye and upper respiratory tract irritation. Formaldehyde is genotoxic and cytotoxic, inducing DNA damage and chromosomal changes [8]. Increased genomic instability from genotoxic chemicals can increase the risk of cancer [9–12]. Formaldehyde is classified as a human carcinogen (Group 1) by the International Agency for Research on Cancer (IARC), based on studies of nasopharyngeal cancer and leukemia [13]. However, studies on the causal relationship between formaldehyde exposure and leukemia development are controversial, with conflicting results [14].

Advances in molecular biology and bioinformatics have led to the development of disciplines that focus on the organization and analysis of large-scale biological data [15, 16]. Toxicogenomics (a combination of toxicology and genomics) is a field that studies genomic responses to xenobiotic exposures [17, 18]. Toxicogenomics provides information on the effects of toxicant exposure on humans, ranging from genetic alterations to disease, based on a genetic profile, with the goal of identifying biomarkers and toxicity mechanisms using high-throughput technologies [19–21]. Many public resources on chemical–gene–disease interactions and other toxicogenomic information can be easily accessed [22]. The macroscopic integration of existing knowledge can result from big data utilization, leading to new perspectives on intricate biological interactions. In this review, we discuss the carcinogenicity of formaldehyde in relation to leukemia through a toxicogenomic approach. Using public and commercial databases, we explore biological networks to better understand the association between formaldehyde exposure and leukemia development.

### Review

#### Carcinogenicity of formaldehyde

In the early 1980s, findings from studies on nasal tumors in rats exposed to formaldehyde provoked concern about its carcinogenicity [23–25]. Diverse chronic and sub-chronic rodent studies provided sufficient evidence that inhalation and oral administration routes of formaldehyde exposure induce cancer [13]. Furthermore, concentration-dependent increases of formaldehyde on tumor incidence and cell proliferation were demonstrated [26, 27]. The association between formaldehyde and the development of cancers was also reported in many epidemiological studies [13]. At the National Cancer Institute (NCI), Hauptmann et al. conducted the largest epidemiological study on occupational exposure to formaldehyde and found a statistically significant increase in death from nasopharyngeal cancer [28]. Based on the comprehensive results of large-scale human and animal studies, the IARC concluded that formaldehyde causes nasopharyngeal cancer and leukemia and is positively associated with sinonasal cancer [13].

Formaldehyde can react with DNA, and in the majority of studies, it displays genotoxicity during mutation tests in vitro and in vivo [29]. Increased DNA damage, micronucleus formation, sister chromatid exchanges, and chromosome aberrations in peripheral lymphocytes and nasal mucosa were observed during human occupational studies on formaldehyde exposure [30–32]. Significant changes in the percentage of B cells, cytotoxic T cells, and natural killer cells were found, and genetic polymorphisms in metabolic and DNA repair genes were associated with increased genetic damages in subjects exposed to formaldehyde [33–35]. Exogenous and endogenous formaldehyde can induce *N*^*2*^-hydroxymethyl-dG adducts [36]. In vitro studies showed induction of DNA–protein crosslinks (DPCs) by formaldehyde exposure in white blood and nasal epithelial cells [37, 38]. In addition, DPCs in white blood cells were higher in workers exposed to formaldehyde than in non-exposed workers [37, 39]. As the early lesions in the process of carcinogenesis, the level of DPCs is considered a biomarker of formaldehyde exposure [37, 39]. These genotoxic effects are the potential carcinogenic mode of action for formaldehyde [5, 27].

#### Association between formaldehyde exposure and leukemia incidence

Although the carcinogenicity of formaldehyde as a consequence of chronic exposure has been indicated [13], the biological mechanisms by which formaldehyde induces cancer are not completely understood. The association between formaldehyde exposure and the occurrence of leukemia is especially disputable. After examining the data from various epidemiological and animal studies, the IARC concluded that there is “strong but not sufficient evidence” that formaldehyde causes leukemia [13]. Three large industrial cohort studies [40–42] notably influenced the interpretation of other studies on formaldehyde exposure and leukemia, and positive associations were observed in the two cohort studies [41, 42]. Coggon et al. investigated a cohort of 14,014 workers at six British factories where formaldehyde was produced or used, and they observed no association [40]. Hauptmann et al. retrospectively analyzed the data from a study undertaken by the NCI that included 25,619 workers at 10 U.S. industrial plants that used or produced formaldehyde [41]. Pinkerton et al. conducted a study for the National Institute for Occupational Safety and Health that included 11,039 workers in three garment plants where formaldehyde resins were used in fabric processing [42]. These three original studies and their updated versions are summarized in Table 1 [40–45]. A review of the recent study findings that included an extended follow-up period after 10 years or more showed that the risk of leukemia tended to decrease. Some case-control studies evaluated the risk of lymphohematopoietic malignancies, but no significant elevations of leukemia risk were found [46–49]. Among these studies, Linos et al. showed a significantly elevated risk for acute myeloid leukemia (AML) among employees in funeral homes and crematories when age and state of residence were adjusted (three exposed cases, odds ratio = 6.7, 95% confidence interval = 1.2–36.2) [47]. Hauptmann et al. also reported a significantly increased risk for myeloid leukemia among funeral industry workers who performed embalming for more than 34 years compared to subjects who performed embalming less than 500 times (14 exposed cases, odds ratio = 3.9, 95% confidence interval = 1.2–12.5, *p* = 0.024) [50]. Other epidemiological studies, meta-analyses, and re-evaluations of previous studies showed inconsistent findings on the association between formaldehyde and leukemia [2, 8, 13, 51–53].

**Table 1:** Summary of large cohort studies about formaldehyde and leukemia

Author	Cohort descriptions	Results (95% CI)	Comments
Coggon et al. [40]	14,014 workers at factories 1941–2000	Leukemia: 31 deaths SMR 0.91 (0.62–1.29) Leukemia: 8 deaths SMR 0.71 (0.31–1.39)	All subjects High exposure ≥2.0 ppm group
Coggon et al. [43]	Update of Coggon et al. 1941–2012	Leukemia: 54 deaths SMR 1.02 (0.77–1.33) Myeloid Leukemia: 36 deaths SMR 1.20 (0.84–1.66) Leukemia: 13 deaths SMR 0.82 (0.44–1.41) Myeloid Leukemia: 8 deaths SMR 0.93 (0.40–1.82)	All subjects High exposure ≥2.0 ppm group
Hauptmann et al. [41]	25,619 workers at factories 1966–1994	Leukemia: 29 deaths RR 2.46 (1.31–4.62) Myeloid Leukemia: 14 deaths RR 3.46 (1.27–9.43)	Peak exposure ≥4.0 ppm group Compared low peak exposure (0.1–1.9 ppm) 35 years of median length of follow-up
Beane Freeman et al. [44]	Update of Hauptmann et al. 1966–2004	Leukemia: 48 deaths RR 1.42 (0.92–2.18) Myeloid Leukemia: 19 deaths RR 1.78 (0.87–3.64)	Peak exposure ≥4.0 ppm group 42 years of median length of follow-up
Pinkerton et al. [34]	11,039 garment workers 1955–1998	Leukemia: 15 deaths SMR 1.92 (1.08–3.17) Myeloid Leukemia: 8 deaths SMR 2.55 (1.10–5.03)	The mean TWA exposure 0.15 ppm Multiple cause mortality from leukemia and myeloid leukemia 10+ years exposure and 20+ years since the first exposure
Meyers et al. [45]	Update of Pinkerton et al. 1955–2008	Leukemia: 23 deaths SMR 1.74 (1.10–2.60) Myeloid Leukemia: 10 deaths SMR 1.90 (0.91–3.50)	Multiple cause mortality from leukemia and myeloid leukemia 10+ years exposure and 20+ years since the first exposure

*CI* Confidence interval, *SMR* Standardized mortality ratio, *RR* Relative risk, *TWA* Time-weighted average

There is debate on the biological plausibility of whether formaldehyde can induce distant-site toxicity, as formaldehyde is rapidly metabolized and highly reactive, and its toxicity is generally limited to the local exposure site [5, 54]. Therefore, it would be valuable to demonstrate the formaldehyde-induced leukemogenic traits, such as 1) exogenous formaldehyde can reach the bone marrow, 2) formaldehyde can induce hematopoietic toxicity, and 3) leukemia occurs in animal models exposed to formaldehyde [29]. Zhang et al. suggested three potential mechanisms for formaldehyde-induced leukemia: direct damage to stem cells in the bone marrow through the blood, damage to hematopoietic stem/progenitor cells circulating in the blood, and damage to primitive pluripotent stem cells present within nasal or oral passages [8]. Considering that formaldehyde and its metabolic pathway exist naturally in all cells, it is likely that formaldehyde toxicity occurs as a result of high concentration exposure that overwhelms normal metabolic capacities [29]. As a basis for the occurrence of distant-site toxicity, several studies were undertaken to estimate increased formaldehyde concentrations in the blood due to formaldehyde exposure. Significant effects of formaldehyde exposure were not observed in the blood of humans exposed to 1.9 ppm for 40 min, rats exposed to 14.4 ppm for 2 h [7], or in rhesus monkeys exposed to 6 ppm for 6 h/day, 5 days/week for 4 weeks [55]. The failure of exogenous formaldehyde to increase formaldehyde levels in the blood decreases the likelihood that formaldehyde directly affects the bone marrow. Other studies used radiolabeled formaldehyde to examine its systemic toxicity in distant sites. Rats exposed to up to 15 ppm of ^14^C- and ^3^H-formaldehyde for 6 h did not show an increase in covalent adducts in the bone marrow [56]. Rats lacking glutathione, required for formaldehyde oxidation, also did not show an increase in covalent adducts in bone marrow following formaldehyde inhalation [57]. In rats and non-human primates, exogenous DNA adducts formed by inhaled ^13^CD_2_-formaldehyde were found only in the nasal epithelium and not in the bone marrow and peripheral blood cells (rats: 10 ppm, 1 or 5 days; 2 ppm, 7–28 days; 15 ppm, 1–4 days; monkeys: 6 ppm, 2 days) [36, 58–60]. These studies concluded that exogenous formaldehyde does not cause distant-site toxicity beyond the portal of entry. Although the exposure periods tend to be short, these results show that Zhang et al.’s hypothesized modes of action of formaldehyde-induced leukemia might be unlikely [14].

In contrast, some animal studies reported an increased incidence of hematological malignancies or toxicity in bone marrow following formaldehyde exposure. The incidence of lymphoma in mice exposed to 14.3 ppm for 2 years was slightly increased (*p* = 0.06), and survival-adjusted undifferentiated leukemia in rats was increased (*p* < 0.0167) [24]. Hematopoietic tumors in high-dose oral-exposed rats [61] and clastogenic and cytogenetic effects on the bone marrow in rats exposed to low concentrations of formaldehyde (0.5 or 1.5 mg/m^3^) were observed [62]. However, there were questions regarding the data reliability of these studies, and other studies contradicted the results [29, 54]. Recent animal studies designed to simulate human occupational exposures reported bone marrow toxicities induced by formaldehyde, suggesting potential toxic mechanisms via oxidative stress. Mice exposed to up to 3 mg/m^3^ formaldehyde (8 h/day for 7 days) by nose-only inhalation exhibited a significant dose-dependent increase in reactive oxygen species and DPCs and a decrease of glutathione in distant organs, including bone marrow [63]. Under a similar exposure condition for 2 weeks, there was a significant decrease in the counts of leucocytes, erythrocytes, and lymphocytes and bone marrow toxicity induced via oxidative stress, inflammation, and apoptosis [64, 65]. Furthermore, whole-body inhalation of 3 mg/m^3^ in mice decreased nucleated bone marrow cells and colony formation from bone marrow stem/progenitor cells, increasing oxidative stress [66, 67]. In mice exposed to formaldehyde (20, 40, and 80 mg/m^3^) for 15 days (2 h/day), bone marrow toxicities (pathological changes, decreased activity of antioxidants, increased micronucleus, DNA damage, and malondialdehyde) and expression changes in Prx, Mpo, Bax, Bcl-2, and Cycs protein were observed [68, 69]. Deficiencies of the genes *Aldh2*, *Adh5*, and *Fancd2*, which detoxify endogenous formaldehyde, led to hematotoxicity and leukemia in mice [70, 71]. Interestingly, studies showing bone marrow toxicity caused by formaldehyde were mainly conducted in mice, an important consideration in light of the interspecies differences in exposure effects [72]. Based on these results, indirect or unknown effects of formaldehyde may cause toxicity at the distant site, including bone marrow. Alterations in the genes related to leukemia development caused by formaldehyde exposure would be key events in converting a hematopoietic stem cell into a leukemic stem cell and subsequent disease development [8, 73].

Inconsistency of results from numerous studies about formaldehyde exposure and leukemia demonstrates the need to consider the effects of individual genetic backgrounds, interspecies differences, and exposure concentration and duration, as the expression of a phenotype can differ between individuals equally exposed to a toxicant [74–77]. In this regard, to help better understand this complexity, we applied a novel approach that utilizes genomic data in order to summarize the association between formaldehyde and leukemia. In the summarizing process, we additionally suggest specific genes as potential biomarker candidates with strong links to formaldehyde exposure and leukemia development.

#### Formaldehyde-related genomic resources

With the development of bioinformatics, approaches that utilize existing data to elucidate biological phenomena are widely used. Biological databases manage diverse data types, such as DNA, RNA, proteins, diseases, pathways, and literature studies [78]. The active use of databases can provide new perspectives on human-related studies, such as biomarker identification, prediction of human health effects, early diagnosis of disease, and drug development [79, 80]. To explore the association between formaldehyde exposure and leukemia through molecular network analysis, we searched formaldehyde-related genes from public and commercial databases.

The Comparative Toxicogenomics Database (CTD) is a publicly available database that provides information about the human health effects of chemicals (http://ctdbase.org/). Its core contents include various chemical–gene–disease interactions manually curated from the literature [22]. These data are not only internally integrated with each other but also with external datasets in order to expand networks and predict novel inferences. Pathway Studio (version 12.3; Elsevier, Netherlands) is a commercially available text mining-based biological network analysis software that enables researchers to explore molecular interactions of diverse biological processes and visualize this information by integrating knowledge from millions of scientific publications [81]. Through the keyword search in CTD and the Pathway Studio database, we retrieved 3927 and 416 formaldehyde-related genes, respectively (accessed 1 Oct 2020). We then identified 122 common genes affected by exogenous formaldehyde exposure in both databases through the examination of the original papers (Supplementary materials).

#### Possibility of oxidative stress-mediated leukemia development

Network-based approaches are widely used to elucidate dynamic biological interactions [82, 83]. The greatest advantage of molecular network analysis is that it can be used to determine interactions among multiple factors that affect a correlation, based on a vast scientific literature, instead of focusing on one-dimensional relationships among a small number of factors. To infer the association between formaldehyde exposure and leukemia incidence, we explored key molecular networks for the 122 common genes using the Pathway Studio software. Pathway Studio presents biological relations through the connectivity (edge) among the entities (nodes), such as genes, cell processes, diseases, or chemicals. The relation is determined using reference sentences extracted from the scientific literature and the number of references [81]. Therefore, our network approach sums up the existing knowledge known between formaldehyde and leukemia.

**Fig. 1:**
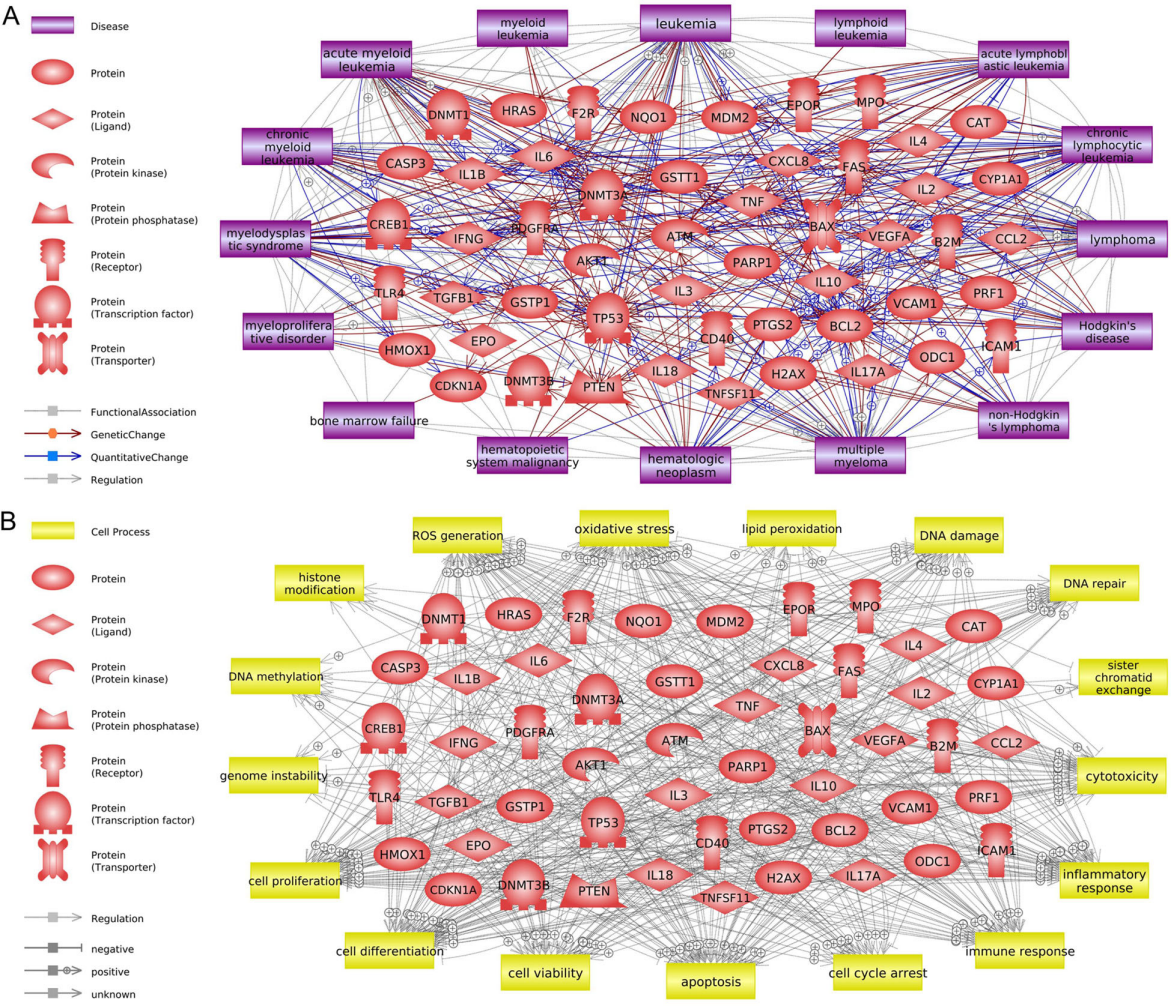
Biological interactions among the formaldehyde-related genes, **a** hematological malignancies, and **b** cell processes. The molecular network analysis was conducted using Pathway Studio software (version 12.3). The relations between genes/proteins and other entities (disease and cell process) were analyzed. The schematic legend is located to the *left*

**Fig. 2:**
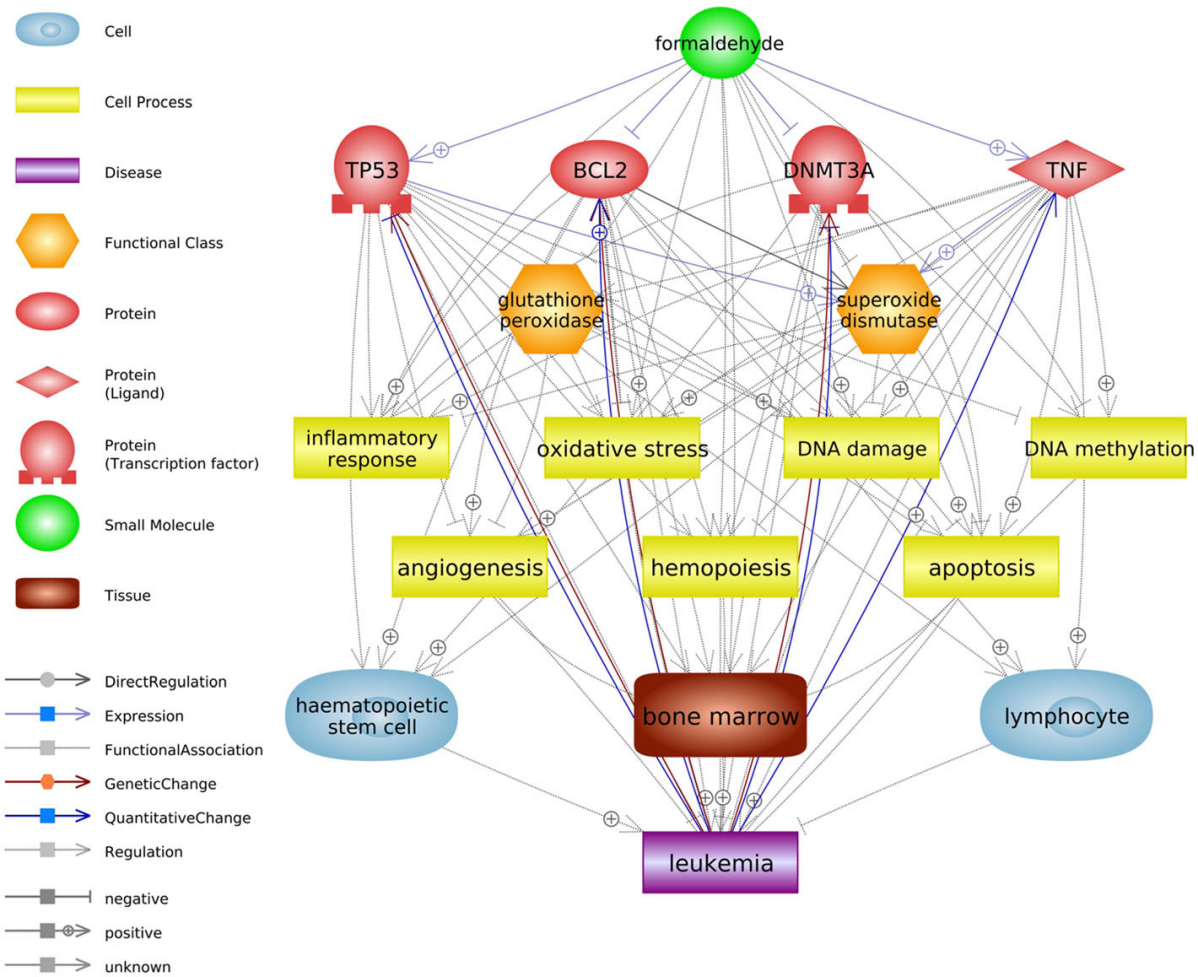
The summarized network of the potential leukemogenic mechanism via oxidative stress. The interactions between selected entities with many associations in previous network analyses and leukemia-related entities were analyzed

The biological interactions of the 122 genes associated with hematological malignancies and cell processes were initially predicted (Fig. 1, Supplementary materials). For the minimal academic credibility, only relations confirmed by more than three references were considered. Subsequently, the criteria of the number of references were adjusted, considering the total connectivity on each entity (i.e., the total amount of relations) in the Pathway Studio database (1 reference per 1000 connectivity) to reduce the bias of analysis from interactions that have been more intensively studied. We selected formaldehyde-related hematological malignancies by referring to epidemiological data in the IARC report published in 2012 [13]. The relation types of “Quantitative Change,” “Genetic Change,” and “Regulation” between genes and diseases were analyzed (Fig. 1a). These genes were associated with many subtypes of leukemia, lymphoma, and myeloma (AML, chronic myeloid leukemia, acute lymphoblastic leukemia, chronic lymphocytic leukemia, Hodgkin’s disease, non-Hodgkin’s lymphoma, myelodysplastic syndrome, etc.). Figure 1b shows that these genes regulate the cell processes associated with the genotoxic and cytotoxic effects of formaldehyde. Our prediction suggests that exposure to formaldehyde increases the generation of reactive oxygen species and induces oxidative stress and DNA damage, resulting in cytotoxicity and an increased cancer risk caused by abnormal cell proliferation and differentiation. Additionally, a detailed review summarized in Fig. 2 was conducted by screening major genes, cell processes, and leukemic diseases with many interactions in the network to determine their influence on the hematopoietic system. We carefully examined the reliability of references regarding the correlation between formaldehyde exposure and other entities to distinguish any inaccurate reference information that the text mining technique could have produced; for example, are the associations negative or positive simply based on the number of references? Do the studies come primarily from one research group? However, our current analysis could not distinguish conflicting interests among research groups. As a result of this detailed literature-based prediction, we hypothesize that formaldehyde can induce the development of leukemia by disturbing the normal differentiation process of hematopoietic stem cells through the induction of dysfunctions in major genes via oxidative stress. Furthermore, it is predicted that formaldehyde could interfere with the function of antioxidant enzymes in the bone marrow and lymphocytes. Alterations of genes *GSTT1* and *GSTP1* that inhibit oxidative stress [84] were associated with leukemia, especially AML. The expression changes in these genes were also identified in the bone marrow of mice that inhaled formaldehyde and rats’ white blood cells, respectively [64, 85]. Abnormal lymphocytes are a major feature of lymphohematopoietic malignancies [86, 87]. To support the reliability of the networks, we categorized the top diseases and biological functions of selected genes in Fig. 1 using Ingenuity Pathway Analysis (Qiagen, Germany), a popular bioinformatics analysis software (Supplementary Table 1). Selected formaldehyde-related genes were associated with cancer and hematological system development and function. Especially, we suggest major genes that are worth considering when attempting to identify the links between formaldehyde exposure and leukemia (Table 2). Several studies reported that formaldehyde induces expression changes in TP53 and BCL2, responsible for regulating apoptotic mechanisms [88–90]. Abnormal apoptosis due to formaldehyde exposure may lead to unregulated self-renewal of hematopoietic stem/progenitor cells [66, 91]. The BAX/BCL2 ratio has clinical significance in leukemias [92]. Furthermore, DNMT3A, which is regulated by TP53, is frequently mutated in AML and other hematological malignancies [93], and a decrease in DNMT3A expression by formaldehyde exposure suggested that formaldehyde has hypomethylation effects [94, 95]. In various leukemic disease studies, TNF expression was increased and associated with poor prognosis [96–98]. Therefore, identifying the changes in major genes in the hematopoietic system caused by prolonged exposure to formaldehyde will be valuable in understanding the leukemogenic mechanism.

**Table 2:** Formaldehyde and leukemia related major gene descriptions

Gene	Functional class	Cell process	Disease
*BCL2*	SOD	Angiogenesis, Apoptosis, DNA damage, Hemopoiesis, Inflammatory response, Oxidative stress	ALL, AML, CLL, CML, Hematologic neoplasm, Hematopoietic system malignancy, Hodgkin’s disease, Leukemia, Lymphoma, Multiple myeloma, Myelodysplastic syndrome, Myeloid leukemia, non-Hodgkin’s lymphoma
*DNMT3A*		Apoptosis, DNA methylation, Hemopoiesis, Inflammatory response	ALL, AML, CLL, CML, Hematologic neoplasm, Hematopoietic system malignancy, Leukemia, Lymphoma, Myelodysplastic syndrome, Myeloid leukemia, Myeloproliferative disorder
*TNF*	GPx, SOD	Angiogenesis, Apoptosis, DNA damage, Hemopoiesis, Inflammatory response, Oxidative stress	ALL, AML, CLL, CML, Hodgkin’s disease, Leukemia, Lymphoma, Multiple myeloma, Myelodysplastic syndrome, non-Hodgkin’s lymphoma
*TP53*	SOD	Angiogenesis, Apoptosis, DNA damage, DNA methylation, Hemopoiesis, Inflammatory response, Oxidative stress	ALL, AML, Bone marrow failure, CLL, CML, Hematologic neoplasm, Hematopoietic system malignancy, Hodgkin’s disease, Leukemia, Lymphoid leukemia, Lymphoma, Multiple myeloma, Myelodysplastic syndrome, Myeloid leukemia, Myeloproliferative disorder, non-Hodgkin’s lymphoma

*SOD* Superoxide dismutase, *ALL* Acute lymphoblastic leukemia, *AML* Acute myeloid leukemia, *CLL* Chronic lymphocytic leukemia, *CML* Chronic myeloid leukemia, *GPx* Glutathione peroxidase

Utilizing a literature-based network approach, we explored qualitative associations between formaldehyde and comprehensive leukemia. It was also predicted that altered gene expression or mutation triggered by oxidative stress because of formaldehyde exposure could disturb the hematopoietic system and lead to an increased risk of malignant hematopoietic diseases. Given that the biological plausibility of distant-site toxicity by formaldehyde inhalation is a key point in elucidating the possibility of leukemogenesis [14], we also examined our toxicogenomic data for genes/proteins that showed activity changes at distant sites following formaldehyde inhalation. Low concentrations of inhaled formaldehyde increased *PDGFRA* and *MDM2* gene expressions in human peripheral lymphocytes of the residents of new apartments [99]. It was also shown that *TXN* gene expression decreased in human blood for subjects under controlled conditions [100]. Gene expression changes in *Gpx3*, *Gstp1*, *Odc1*, *Polr2a*, *Ptgs1*, and *Rps6ka5* were identified in white blood cells of rats exposed to 2 ppm formaldehyde [85]. In addition, we identified gene expression changes (*Atm*, *Epo*, *Cyp1a1*, and *Gstt1*) and protein expression changes (Csf2ra, Epo, Epor, Bax, Bcl2, Mpo, and Prx2) in the bone marrow of mice that inhaled formaldehyde [64, 66–69]. The cytokine levels of TNF-α and IL-1β were increased in the bone marrow of formaldehyde-exposed mice [64]. The polymorphisms in *GSTP1* and *PARP1* genes were related to increased genetic damage in peripheral blood lymphocytes of formaldehyde-exposed subjects [34]. Although not all genes in our toxicogenomic data reflect distant-site toxicity, some genes associated with leukemic diseases showed altered expression at distant sites following formaldehyde exposure. Based on these findings, indirect or unknown leukemia-inducing mechanisms caused by formaldehyde on the hematopoietic system cannot be ruled out.

## Conclusions

In this review, we explored the controversial association between exposure to the carcinogen formaldehyde and the incidence of leukemia. Although there are inconsistent results on this topic, recent studies reported the bone marrow or hematopoietic toxicity by formaldehyde [65–67]. We analyzed biological networks among formaldehyde-related genes retrieved from public and commercial databases to help understand the association between formaldehyde and leukemia. Our literature-based prediction suggests a potential leukemia-inducing mechanism of formaldehyde via oxidative stress, as well as major genes associated with formaldehyde and leukemia. Validation of these genes should be performed in further studies. To better understand the leukemogenicity of formaldehyde, reproducible experiments that determine the causality are needed. Important factors, such as individual genetic backgrounds, interspecies differences, and exposure degree, should be considered. Further studies that correctly evaluate the distant-site toxicity utilizing well-designed genomic data to simulate prolonged occupational exposure will also be needed. Nevertheless, the possibility of other perspectives, such as aberrant activation of major genes and signaling pathways, is also worth considering. Our approach can be used to complement experimental data for elucidating the effects of genetic factors and can be applied in the identification of new mechanisms and biomarkers.

## Supplementary Information

**Additional file 1.**

**Additional file 2.**

## Data Availability

Not applicable.

## Abbreviations

ALL: Acute lymphoblastic leukemia
AML: Acute myeloid leukemia
CI: Confidence interval
CLL: Chronic lymphocytic leukemia
CML: Chronic myeloid leukemia
CTD: Comparative Toxicogenomics Database
GPx: Glutathione peroxidase
IARC: International Agency for Research on Cancer
IPA: Ingenuity Pathway Analysis
NCI: National Cancer Institute
OR: Odds ratio
RR: Relative risk
SMR: Standardized mortality ratio
SOD: Superoxide dismutase
TWA: Time-weighted average
