# Supplementary material for: Formaldehyde exposure and leukemia risk: a comprehensive review and network-based toxicogenomic approach
Source: Genes Environ. 2021 Apr 12;43:13. doi: 10.1186/s41021-021-00183-5 (PMC8042688; doi:10.1186/s41021-021-00183-5)
Supplement: Supplementary file 1 — Additional file 1. [file 41021_2021_183_MOESM1_ESM.docx]

Toxicogenomic analysis process

We downloaded entire formaldehyde-related gene data from the Pathway Studio and CTD. We manually confirmed reference sentences and original articles extracted by text-mining and then, identified 122 common genes affected by exogenous formaldehyde exposure in both databases. To analyze biological interactions between these genes and leukemia, formaldehyde-related hematological malignancies were selected by referring to IARC's 2012 report. Furthermore, cell processes associated with genotoxicity and cytotoxicity known to be induced by formaldehyde were selected in the Pathway Studio database. The network analyses were conducted using the "Direct Interactions" algorithm of the Pathway Studio based on the minimum number of references (≥3). To reduce the bias of analysis from interactions that have been more intensively studied, the criteria of the number of references were differentially adjusted, considering the total amount of relations* on each entity in the Pathway Studio database. The top four major genes highly associated with hematological malignancies were selected considering the total number of references and connectivity (lines) of each relation in the network**. The current Pathway Studio's system provides information between the same or other types of entity (e.g., gene–gene, disease–disease, gene–disease, cell process–disease). However, it does not provide relations between cell processes. Therefore, even though some interactions are well-known, such as the interaction between oxidative stress and DNA damage, there is no line between cell processes on the networks.

* Relation between entities means the biological interaction extracted from the scientific articles (for further details, please refer to the Quick Reference Guide: <https://service.elsevier.com/app/answers/detail/a_id/2683/supporthub/pathway-webmammal/>). In addition, each relation is expressed as a line (connectivity) on the network and specifies the number of references supporting this interaction.

** Summary of network information based on Fig 1A.

| Gene | Total number of references | Connectivity | Total number of references  / Connectivity |
| --- | --- | --- | --- |
| *TP53* | 3045 | 31 | 98.23 |
| *TNFSF11* | 104 | 2 | 52.00 |
| *BCL2* | 801 | 26 | 30.81 |
| *DNMT3A* | 614 | 22 | 27.91 |
| *ATM* | 447 | 17 | 26.29 |
| *VEGFA* | 346 | 14 | 24.71 |
| *IL6* | 441 | 19 | 23.21 |
| *TNF* | 433 | 21 | 20.62 |
| *AKT1* | 122 | 6 | 20.33 |
| *IL2* | 215 | 12 | 17.92 |
| *EPO* | 89 | 5 | 17.80 |
| *PTEN* | 302 | 17 | 17.76 |
| *CD40* | 54 | 4 | 13.50 |
| *FAS* | 188 | 14 | 13.43 |
| *MDM2* | 125 | 10 | 12.50 |
| *IL3* | 124 | 10 | 12.40 |
| *IFNG* | 146 | 12 | 12.17 |
| *IL10* | 175 | 15 | 11.67 |
| *PRF1* | 44 | 4 | 11.00 |
| *PDGFRA* | 71 | 7 | 10.14 |
| *TGFB1* | 91 | 9 | 10.11 |
| *IL4* | 20 | 2 | 10.00 |
| *DNMT1* | 29 | 3 | 9.67 |
| *IL17A* | 28 | 3 | 9.33 |
| *CREB1* | 18 | 2 | 9.00 |
| *HRAS* | 17 | 2 | 8.50 |
| *HMOX1* | 33 | 4 | 8.25 |
| *B2M* | 49 | 6 | 8.17 |
| *BAX* | 55 | 7 | 7.86 |
| *CXCL8* | 62 | 8 | 7.75 |
| *GSTT1* | 69 | 9 | 7.67 |
| *IL1B* | 46 | 6 | 7.67 |
| *NQO1* | 53 | 7 | 7.57 |
| *CDKN1A* | 28 | 4 | 7.00 |
| *ODC1* | 7 | 1 | 7.00 |
| *EPOR* | 13 | 2 | 6.50 |
| *GSTP1* | 58 | 10 | 5.80 |
| *H2AX* | 23 | 4 | 5.75 |
| *CYP1A1* | 11 | 2 | 5.50 |
| *IL18* | 22 | 4 | 5.50 |
| *CCL2* | 10 | 2 | 5.00 |
| *VCAM1* | 10 | 2 | 5.00 |
| *PTGS2* | 19 | 4 | 4.75 |
| *ICAM1* | 9 | 2 | 4.50 |
| *PARP1* | 9 | 2 | 4.50 |
| *F2R* | 4 | 1 | 4.00 |
| *TLR4* | 4 | 1 | 4.00 |
| *CAT* | 7 | 2 | 3.50 |
| *MPO* | 7 | 2 | 3.50 |
| *DNMT3B* | 10 | 3 | 3.33 |
| *CASP3* | 3 | 1 | 3.00 |
