# Supplementary material for: Formaldehyde exposure and leukemia risk: a comprehensive review and network-based toxicogenomic approach
Source: Genes Environ. 2021 Apr 12;43:13. doi: 10.1186/s41021-021-00183-5 (PMC8042688; doi:10.1186/s41021-021-00183-5)
Supplement: Supplementary file 2 — Additional file 2. [file 41021_2021_183_MOESM2_ESM.docx]

Supplementary table 1. Top diseases and bio functions in IPA core analysis result.

| Diseases and Disorders | *P* value range | No. of genes |
| --- | --- | --- |
| Organismal Injury and Abnormalities | 1.80E-21 – 3.14E-43 | 51 |
| Cancer | 1.64E-21 – 1.48E-41 | 51 |
| Tumor Morphology | 5.99E-22 – 4.76E-39 | 41 |
| Inflammatory Response | 1.22E-21 – 5.36E-38 | 47 |
| Hematological Disease | 1.64E-21 – 1.26E-36 | 50 |
| Physiological System Development and Function | *P* value range | No. of genes |
| Hematological System Development and Function | 1.31E-21 – 2.28E-45 | 49 |
| Tissue Morphology | 1.06E-21 – 2.28E-45 | 50 |
| Organismal Survival | 3.67E-33 – 1.63E-41 | 49 |
| Lymphoid Tissue Structure and Development | 1.31E-21 – 2.42E-41 | 46 |
| Cardiovascular System Development and Function | 2.80E-22 – 1.14 E-38 | 41 |

The *P* value range indicates the range from lowest to the highest *P* value of disease or function included in each category. No. of genes indicates the total number of genes involved in disease or function of each category. IPA, Ingenuity Pathway Analysis.
